# Factors associated with fear of childbirth in a subsequent pregnancy: a nationwide case–control analysis in Finland

**DOI:** 10.1186/s12905-023-02185-7

**Published:** 2023-01-24

**Authors:** Matias Vaajala, Rasmus Liukkonen, Ilari Kuitunen, Ville Ponkilainen, Ville M. Mattila, Maiju Kekki

**Affiliations:** 1 Faculty of Medicine and Life Sciences, University of Tampere, Tampere, Finland; 2 Department of Pediatrics, Mikkeli Central Hospital, Mikkeli, Finland; 3 Institute of Clinical Medicine and Department of Pediatrics, University of Eastern Finland, Kuopio, Finland; 4 Department of Surgery, Central Finland Central Hospital Nova, Jyväskylä, Finland; 5 Department of Orthopaedics and Traumatology, Tampere University Hospital Tampere, Tampere, Finland; 6 Department of Obstetrics and Gynaecology, Tampere University Hospital, Tampere, Finland; 7 Center for Child, Adolescent and Maternal Health Research, Faculty of Medicine and Health Technology, Tampere University, Tampere, Finland

**Keywords:** Cesarean section, Fear of childbirth, Obstetrics

## Abstract

**Background:**

Fear of childbirth can develop due to the concerns or adverse maternal or foetal outcomes experienced in a previous pregnancy. The aim of this study was to examine the main risk factors associated with the development of fear of childbirth during subsequent pregnancies and deliveries.

**Methods:**

In this case–control study, data from the National Medical Birth Register were used to evaluate the events in previous pregnancies that were potential risk factors for fear of childbirth in subsequent pregnancies. The first and second pregnancies of women registered during our study period (2004–2018) were included. The exposure variable was delivery mode, obstetric challenge or adverse neonatal outcomes during the first pregnancy. The outcome was the development of FOC during the second pregnancy. Adjusted odds ratios with 95% CIs were used for comparison.

**Results:**

A total of 13 064 pregnancies were included in the case group and 195 351 in the control group. Previous emergency caesarean section was the strongest risk factor for the development of FOC in the second pregnancy (adjusted odds ratio 5.27, CIs 4.83–5.75). In addition, unplanned CS (adjusted odds ratio 3.93, CIs 3.77–4.10) and vacuum delivery (adjusted odds ratio 1.69, CIs 1.61–1.77) also increased the odds of fear of childbirth. Of the obstetric complications, third- or fourth-degree tear of the perineum was the strongest risk factor (adjusted odds ratio 2.99, CIs 2.69–3.31), followed by shoulder dystocia (adjusted odds ratio 2.82, CIs 2.16–3.62). Neonatal mortality also increased the odds for the development of FOC (adjusted odds ratio 2.17, CIs 1.77–2.64).

**Conclusion:**

The main risk factors for the development of fear of childbirth in the second pregnancy were previous fear of childbirth, unplanned CS, vacuum delivery, perineal tear or shoulder dystocia. The results of this study can be used in a clinical setting to improve the prevention of fear of childbirth.

**Supplementary Information:**

The online version contains supplementary material available at 10.1186/s12905-023-02185-7.

## Background

Fear of childbirth (FOC) is a common obstetrical challenge affecting the health of women [1]. Higher socioeconomic status, advanced maternal age and depression, as well as previous operative deliveries (vacuum or emergency caesarean delivery), are all predictive factors for FOC [2, 3]. However, studies conducted in Finland have revealed that psychosocial factors, such as anxiety, neuroticism, depression, low self-esteem and lack of social support, also play an important role in the development of FOC [4]. In the last study of the incidence of FOC conducted in Finland in 2014, the prevalence of FOC increased from 1.1 to 3.6% in nulliparous women and from 1.5% to 7.8% in multiparous women between 1997 and 2010 [3]. High rates of FOC have also been reported in other Nordic countries. According to a study in the Swedish population, the prevalence of intense FOC was 15.8% and very intense FOC 5.7% [5]. Moreover, in a study made in Norway, 12% of the participants reported FOC [6].

According to a study using a cohort of 100 women with severe FOC, emergency caesarean and vacuum extraction during the women’s first delivery were associated with a secondary fear of delivery during the second pregnancy [7]. A Finnish questionnaire study of 1400 women in 2008 found that severe fear of childbirth was more common in nulliparous women in later pregnancy and in those women who had undergone previous caesarean section or vacuum extraction [8]. To the best of our knowledge, the effects of the events that took place in the previous delivery on the risk of the development of FOC in a subsequent pregnancy has not previously been studied in a nationwide setting. Based on our hypothesis, an adverse outcome during a previous delivery might be one of the strongest predictive factors for the development of FOC. The aim of this study is, therefore, to examine the main risk factors in a previous pregnancy or delivery that might cause FOC in the next pregnancy.

## Materials and methods

In this nationwide retrospective register-based case–control study, data from the National Medical Birth Register (MBR) were used to evaluate the events that took place in a previous pregnancy that may have caused FOC in a subsequent pregnancy. The MBR is maintained by the Finnish Institute for Health and Welfare (THL). The register was established in 1987, and subsequently renewed in 1990, 1996, 2004, and 2017. The goal of the register is to collect data for statistics and research and to develop reproductive health in Finland [9]. The study period was from 1 January 2004 to 31 December 2018.

The MBR contains data on pregnancies, delivery statistics and the perinatal outcomes of all births with a birthweight of ≥ 500 g or a gestational age of ≥ 22^+0^ weeks. The MBR has high coverage and quality (the current coverage is nearly 100%) [10, 11]. The variables included in the MBR are routinely collected in the maternity clinics using a maternity counseling card or in the maternity hospital [9]. All mothers who had their first and second pregnancies registered in the MBR during our study period were included. The study design using the first and second pregnancies is based upon a hypothesis, that maternal FOC in 2nd pregnancy might be associated with some events in the previous pregnancy. All later pregnancies were excluded. The second pregnancies were divided into two groups, a case group and a control group, based on the diagnosed maternal FOC registered in the MBR. In Finland, all women are asked about their fears of childbirth during antenatal visits. Those women who experience a significant FOC, but do not receive enough help during the antenatal visits to women and child welfare clinics and/or have requested caesarean section (CS) due to FOC, are referred to secondary / tertiary maternity clinic. FOC is diagnosed if it is manifested and dealt with during a maternity care visit with a physician or specialized midwife. In the present study, FOC was defined according to the International Classification of Diseases 10th revision code (ICD-10) code O99.80 established in 1997. The registering of FOC in the MBR started in 2004. In the present study, women with diagnosed FOC in their second pregnancy formed the case group and women without a diagnosis formed the control group.

**Fig. 1:**
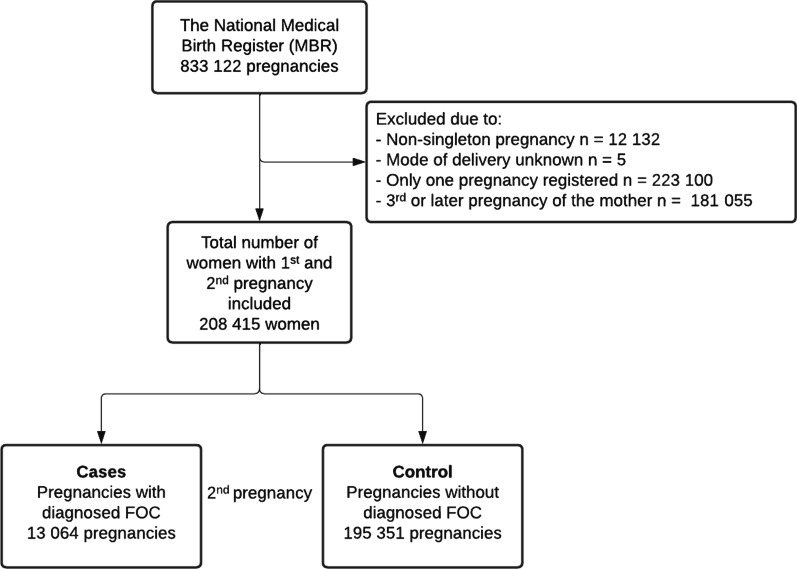
Flowchart depicting the process used to divide the study population into groups

Pregnancies with unknown mode of delivery (n = 5, none of which had diagnosed maternal FOC) and non-singleton pregnancies (n = 12,132) were excluded from the analysis. In addition, women who had only one pregnancy registered during our study period (n = 223,100) or who had a third or later pregnancy (n = 181,055) were excluded. A total of 208,405 women with a first and second pregnancy registered in the MBR met the inclusion criteria. The formation of the study groups is shown as a flowchart (Fig. 1).

## Statistics

Continuous variables were interpreted as means with standard deviations (SDs) or as a median with an interquartile range (IQR) based on the distribution of the data. The categorical variables are presented as absolute numbers and percentages with 95 confidence intervals (CIs). The CIs for rates were calculated using Poisson regression. The multivariable logistic regression model was used to assess the development of FOC during the second pregnancy that was caused by events that took place in the first pregnancy. Women with diagnosed FOC in the second pregnancy were compared to the control group, which consisted of all women without a diagnosis of FOC. The exposure variable was delivery mode (emergency CS, vacuum delivery, unplanned CS), obstetric challenge (uterine curettage, manual placenta removal, third- or fourth-degree tear of the perineum, shoulder dystocia or adverse neonatal outcomes (perinatal mortality, neonatal intensive care, very or extremely preterm delivery) in the first pregnancy. The outcome was the development of FOC during the second pregnancy. Adjusted odds ratios (aORs) with 95% CIs between the groups were compared. The model was adjusted by previously recognized potential confounders that are known to cause FOC in a second pregnancy: maternal age, gestational diabetes and weight of the neonate [12–15]. The model was created separately with women who had FOC in the first pregnancy both included and excluded. Adjustments were made by choosing the variables for a multivariable model using directed acyclic graphs (DAG) constructed using the free online software DAGitty (dagitty.net) [16]. The variables included in the DAG were chosen based on known risk factors and by hypothesized causal pathways (Additional file 1: Figure S1). The results of this study are reported according to the STROBE guidelines [17]. Statistical analyses were performed using R version 4.0.3 for Windows (R Foundation for Statistical Computing, Vienna, Austria).

## Ethics

Both the National Medical Birth Register (MBR) and the Care Register for Healthcare had the same unique pseudonymised identification number for each patient. The pseudonymisation was made by the Finnish data authority Findata, and the authors did not have access to the pseudonymisation key, as it is maintained by Findata. In accordance with Finnish legislation, no informed written consent was required because of the retrospective register-based study design and because the patients were not contacted. Permission for the use of this data was granted by FINDATA after evaluation of the study protocol (Permission number: THL/1756/14.02.00/2020).

## Results

Of the 208 415 women included in this study, 193 454 women had no diagnosed FOC in either their first or second pregnancies. A total of 11 355 women did not have FOC in their first pregnancy, but it developed in their second pregnancy. A further 1897 women developed FOC during their first pregnancy, but not during their second pregnancy. In total, 1709 women developed FOC in both pregnancies. Of those women who had diagnosed FOC in their first pregnancy, but not in their second, 58.6% (CI 55.2–62.1) had spontaneous vaginal delivery and 18.1% (CI 16.2–20.1) had elective CS. Of those women who had FOC in both pregnancies, only 18.9% (CI 16.9–21.1) had spontaneous vaginal delivery in their first pregnancy, whereas over half of the women (50.7%, CI 47.4–54.2) had elective CS as a mode of delivery (Table 1).

**Table 1:** Description on the fear of childbirth (FOC) variable and mode of deliveries variable in 1st and 2nd pregnancies

	Case group FOC in 2nd pregnancy		Control group No FOC in 2nd pregnancy	
Total number of patients	13,064		193,454	

	n	% (95% CI)	n	% (95% CI)
FOC in first pregnancy	1709	13.1 (12.5–13.7)	1897	14.5 (13.9–15.2)
*Delivery mode in first pregnancy*				
Spontaneous vaginal	323	18.9 (16.9–21.1)	1111	58.6 (55.2–62.1)
Instrumental vaginal	150	8.8 (7.4–10.3)	240	12.7 (11.1–14.4)
Unplanned caesarean section	346	20.3 (18.2–22.5)	187	9.9 (8.5–11.4)
Emergency cesarean section	23	1.3 (0.9–2.0)	7	0.4 (0.1–0.8)
Elective caesarean section	867	50.7 (47.4–54.2)	343	18.1 (16.2–20.1)
No FOC in first pregnancy	11 355	86.9 (85.3–88.5)	191 557	99.0 (98.6–99.5)
*Delivery mode in first pregnancy*				
Spontaneous vaginal	3537	31.2 (30.1–32.2)	131 465	68.6 (68.3–69.0)
Instrumental vaginal	2719	24.0 (23.1–24.9)	29 956	15.6 (15.5–15.8)
Unplanned caesarean section	3688	32.5 (31.4–33.5)	19 861	10.4 (10.2–10.5)
Emergency cesarean section	699	6.2 (5.7–6.6)	2336	1.2 (1.2–1.3)
Elective caesarean section	712	6.3 (5.8–6.7)	7939	4.1 (4.1–4.2)

*CI* confidence interval

In the case group, women were older at the time of the second pregnancy (mean 31.2 vs 29.8) when compared to the control group. Furthermore, a higher rate of women had diagnosed gestational diabetes in the case group when compared to the control group (17.8%, CI 17.1–18.5 vs 12.9%, CI 12.8–13.1). Also, a higher rate of women had FOC in their previous pregnancy in the case group than in the control group (13.1%, CI 12.5–13.7% vs 1.0%, CI 0.9–1.0%) (Table 1).

When comparing the preceding pregnancies of women in the case and control groups, a markedly lower rate of women in the case group had spontaneous vaginal delivery (30.0%, CI 28.6–30.5 vs 68.8%, CI 68.5–69.2). However, a higher rate of women in the case group had vacuum delivery (21.3%, CI 20.6–22.1 vs 14.6%, CI 14.4–14.8), unplanned CS (30.9%, CI 29.9–31.9 vs 10.3%, CI 10.1–10.4), emergency CS (5.5%, CI 5.1–5.9 vs 1.2%, CI 1.2–1.2) or elective CS (12.1%, CI 11.5–12.7 vs 4.2%, CI 4.1–4.3) than women in the control group. Uterine curettage, manual placenta removal, third- or fourth-degree tears of the perineum and shoulder dystocia were all more common in the case group than in the control group. The rate for of women spending time in the neonatal intensive care unit after vaginal delivery was more common in the case group than in the control group (Table 2).

**Table 2:** Background information on the study groups

Total (N)	Case group		Control group	
	13,064		195,351	
	n	% (95% CI)	n	% (95% CI)
*Previous pregnancy*				
FOC	1709	13.1 (12.5–13.7)	1897	1.0 (0.9–1.0)
*Mode of delivery in first pregnancy*				
Spontaneous vaginal	3861	30.0 (28.6–30.5)	134 462	68.8 (68.5–69.2)
Vacuum	2787	21.3 (20.6–22.1)	28 485	14.6 (14.4–14.8)
Breech or forceps	82	0.6 (0.5–0.8)	1721	0.9 (0.8–0.9)
Unplanned caesarean section	4034	30.9 (29.9–31.9)	20 050	10.3 (10.1–10.4)
Emergency caesarean section	722	5.5 (5.1–5.9)	2343	1.2 (1.2–1.2)
Elective caesarean section	1579	12.1 (11.5–12.7)	8282	4.2 (4.1–4.3)
*Obstetric challenges*				
Uterine curettage	167	1.3 (1.1–1.5)	1581	0.8 (0.8–0.9)
Manual placenta removal	274	2.1 (1.9–2.4)	2945	1.5 (1.5–1.6)
Third- or fourth-degree tears of the perineum	458	3.5 (3.2–3.8)	2539	1.3 (1.3–1.4)
Shoulder dystocia^a^	74	0.6 (0.4–0.7)	425	0.2 (0.2–0.2)
Adverse neonatal outcomes				
Very or extremely preterm delivery (< 31 + 6 weeks)	74	0.6 (0.4–0.7)	1147	0.6 (0.6–0.6)
Need for intensive care unit^a^	1079	8.3 (7.8–8.8)	16 022	8.2 (8.1–8.3)
Neonatal mortality^b^	112	0.9 (0.7–1.0)	899	0.5 (0.4–0.5)
*The second pregnancy*				
Age (mean; SD)	31.2 (4.8)		29.8 (4.8)	
Smoking status smoker	1524	11.7 (11.1–12.3)	20 988	10.7 (10.6–10.9)
Maternal BMI (kg/m^2^) (mean; SD)	25.1 (5.2)		24.5 (4.9)	
Maternal gestational diabetes	2321	17.8 (17.1–18.5)	25 255	12.9 (12.8–13.1)
Neonatal weight, grams (mean; SD)	3620 (459)		3591(515)	
Induction of labour	2495	19.1 (18.4–19.9)	33 410	17.1 (16.9–17.3)
Length of pregnancy (week + day) (mean; SD)	39 + 4 (8)		39 + 6 (6)	
preterm (< 37 gestational weeks)	258	2.0 (1.7–2.2)	7046	3.6 (3.5–3.7)

Women with FOC (case group) were compared women without FOC (control group) in 2nd pregnancy

*CI* confidence intervals

^a^Only in vaginal deliveries because in caesarean section most neonates need intensive care

^b^Includes stillbirth and those who died during the first week

FOC in the first pregnancy increased the odds for FOC in second pregnancy the most (aOR 14.9, CI 13.9–16.0). Of the delivery modes, previous emergency CS was the strongest risk factor for the development of FOC during the second pregnancy (aOR 5.27, CI 4.83–5.75) when women with FOC in the first pregnancy were excluded. Moreover, the odds for the development of FOC increased markedly after unplanned CS (aOR 3.93, CI 3.77–4.10) and vacuum delivery (aOR 1.69, CI 1.61–1.77) when women who had FOC during their first pregnancy were excluded.

From the obstetric challenges, third- or fourth-degree tears of the perineum was the strongest risk factor (aOR 2.99, CI 2.69–3.31) for the development of FOC in the second pregnancy, followed by shoulder dystocia (aOR 2.82, CI 2.16–3.62) and uterine curettage or manual placenta removal (aOR 1.52, CI 1.36–1.69) when patients with FOC in their first pregnancy were excluded. Of the adverse neonatal outcomes, neonatal mortality increased the odds for the development of FOC the most (aOR 2.17, CI 1.77–2.64). The odds for FOC were higher after vaginal deliveries requiring neonatal intensive care (aOR 1.12, CI 1.05–1.19), but this increase was relatively low when compared to the other risk factors (Table 3).

**Table 3:** Adjusted odds ratios (ORs) with 95% confidence intervals (Cis) for the event of the development of maternal fear of childbirth (FOC) in the 2nd pregnancy

	Development of FOC in the 2nd pregnancy
*Event in the first pregnancy*	
Women with FOC in the first pregnancy included (n = 3606)	aOR* (CI)
FOC in the first pregnancy	14.9 (13.9–16.0)
*Delivery mode*	
Vacuum delivery	1.51 (1.45–1.58)
Emergency caesarean section	4.71 (4.31–5.13)
Unplanned caesarean section	3.65 (3.51–3.80)
*Obstetric challenge*	
Very or extremely preterm delivery (< 31 + 6 weeks of gestation)	0.97 (0.75–1.21)
Uterine curettage or manual placenta removal	1.38 (1.24–1.54)
Third- or fourth-degree tear of the perineum	2.68 (2.41–2.96)
Shoulder dystocia*	2.69 (2.08–3.42)
*Adverse neonatal outcome*	
Neonatal intensive care (in vaginal delivery)	1.10 (0.95–1.08)
Perinatal mortality**	1.89 (1.54–2.30)
*Women with FOC in the first pregnancy excluded*	
*Delivery mode*	
Vacuum delivery	1.69 (1.61–1.77)
Emergency caesarean section	5.27 (4.83–5.75)
Unplanned caesarean section	3.93 (3.77–4.10)
*Obstetric challenge*	
Uterine curettage or manual placenta removal	1.52 (1.36–1.69)
Third- or fourth-degree tear of the perineum	2.99 (2.69–3.31)
*Shoulder dystocia*	2.82 (2.16–3.62)
Adverse neonatal outcome	
Neonatal intensive care (in vaginal delivery)	1.12 (1.05–1.19)
Neonatal mortality*	2.17 (1.77–2.64)
Very or extremely preterm delivery (< 31 + 6 weeks of gestation)	1.12 (0.88–1.42)

*Adjusted by maternal age, diagnosed gestational diabetes and weight of the neonate in the second pregnancy

**Only in deliveries registered as vaginal (spontaneous vaginal or instrumental vaginal)

***Includes stillbirth and those who died during the first week

## Discussion

The main finding of the study was that the strongest risk factors during the first pregnancy for the development of FOC in the subsequent pregnancy were pre-existing FOC and the turning of trial of labour into unplanned or emergency CS. Furthermore, complications during labour, such as third- or fourth-degree tears of the perineum and shoulder dystocia, markedly increased the odds for FOC. Adverse neonatal outcomes had less of an effect on the development of FOC. Indeed, among those patients with FOC in the first pregnancy, spontaneous vaginal delivery decreased the rate of FOC in the second pregnancy.

The odds ratio for the development of FOC after emergency CS was over 5-times higher, and nearly 4-times higher after unplanned CS**.** In 1999, a study with a similar study design, but with a small population of only 100 patients with FOC, investigated the effects of previous delivery mode on the risk of FOC. The study revealed that the risk of FOC after emergency CS was nearly 27-times higher [7], which is much higher than our results. The main reason behind the increased odds for FOC after unplanned or emergency CS is most likely the fear of repeat challenges or complications during childbirth, as they are indications for converting trial of labour into CS. According to the previous literature, presumed foetal compromise and prolonged labour remained the main indications for unplanned and emergency cesareans [18]. A 1998 study examining the feelings of women towards emergency CS found that the decision to undertake a caesarean section brought with it a feeling of relief [19]. However, this feeling was soon replaced by fear as the operation approached [19]. The thoughts of the women centred around the impending delivery and operation until after the event, when the new born baby occupied their attention and happiness predominated [19]. According to another study, women who underwent unplanned CS or instrumental delivery experienced more general mental distress and post-traumatic stress than women who underwent normal vaginal delivery or elective CS [20]. Therefore, previous unplanned mode of delivery might be one explanation for the development of FOC. However, the exact reason for the development of FOC remains unclear due to the crude nature of our data. Further, it remains unclear whether the possible cause of FOC in the previous pregnancy was the delivery mode itself or the factors leading to the mode of delivery.

Complications during childbirth, such as shoulder dystocia and third- or fourth-degree tears of the perineum, markedly increased the odds for the development of FOC. Indeed, the odds for the development of FOC was nearly 3-times higher after these complications. The increased odds for developing FOC after third- or fourth-degree perineum tear was an expected result, as it is known that these events can have physical and psychological consequences. In some cases, women may experience social isolation and marginalisation due to their ongoing symptomatology [21]. In addition, the injury can be painful for the mother [22]. It is also known that a previous tear of the perineum increases the risk for the recurrence of perineal tear [22], which might be one further reason behind the development of FOC. However, it should be acknowledged that delivery complications, such as shoulder dystocia and perineal tear, can both occur during the same pregnancy. Therefore, the exact cause of any future FOC cannot be deduced based on our data.

Even though complications, such as shoulder dystocia and perineal tear, can also occur in pregnancies with spontaneous vaginal delivery, our results reveal that those women with diagnosed FOC in the first pregnancy who had a spontaneous vaginal delivery had a notably lower rate of FOC in the subsequent pregnancy, indicating that spontaneous vaginal deliveries might be associated with a successful course of childbirth. Based on our data, a successful vaginal delivery is a strong factor for the disappearance of FOC. As psychological support is generally useful in preventing FOC [23], psychological support should also be offered after a complicated delivery to prevent secondary FOC.

Interestingly, adverse neonatal outcomes had less of an effect on the odds for the development of FOC than alternate delivery methods or complications during childbirth. The increase after neonatal intensive care was truly restrained when compared to the effect of delivery methods and complications during childbirth. Moreover, the clinical importance is non-existent, as it can be associated with other factors, such as complications during childbirth. The odds for the development of FOC was 2-times higher after perinatal mortality. However, the rate of perinatal mortality in Finland is among the lowest globally, and the number of patients with neonatal mortality was extremely low [24], resulting in increased imprecision in the estimates.

The strengths of our study are the large nationwide register data used and the long study period, which allowed us to analyse the rates of FOC using a large study population. The register data used in our study are routinely collected in structured forms using national instructions, which ensures good coverage (over 99%) and reduces possible reporting and selection biases.

The main limitation of this study is that the severity of the FOC is unknown because, at present, there is no uniform criteria or definitions for FOC. Generally, FOC is defined as anxiety and fear of pregnancy, childbirth or the parenting of a child that impair daily wellbeing. FOC takes different forms in different women and may manifest as physical complaints, nightmares and difficulties to concentrate [25].

## Conclusion

The main risk factors for the development of FOC in the second pregnancy were previous FOC, unplanned emergency or unplanned CS, vacuum delivery, third- or fourth-degree tears of the perineum or shoulder dystocia. However, the effects of adverse neonatal outcomes had less of an effect on the development of FOC. The findings of this study are useful in a clinical setting to improve the prevention of FOC. Psychological support should also be offered after a complicated delivery to prevent secondary FOC.

## Supplementary Information

**Additional file 1**. Directed acyclic graph (DAG).

## Data Availability

The data that support the findings of this study are available from Findata, but restrictions apply to the availability of these data, which were used under license for the current study, and so are not publicly available. Data are however available from the authors upon reasonable request and with permission of Findata (url Findata.fi, email info@Findata.fi). Corresponding author (email matias.vaajala@tuni.fi) can be contacted for the data with a reasonable request.
