# Supplementary material for: Factors associated with fear of childbirth in a subsequent pregnancy: a nationwide case–control analysis in Finland
Source: BMC Womens Health. 2023 Jan 24;23:34. doi: 10.1186/s12905-023-02185-7 (PMC9872275; doi:10.1186/s12905-023-02185-7)
Supplement: Supplementary file 1 — Additional file 1. Directed acyclic graph (DAG). [file 12905_2023_2185_MOESM1_ESM.pdf]

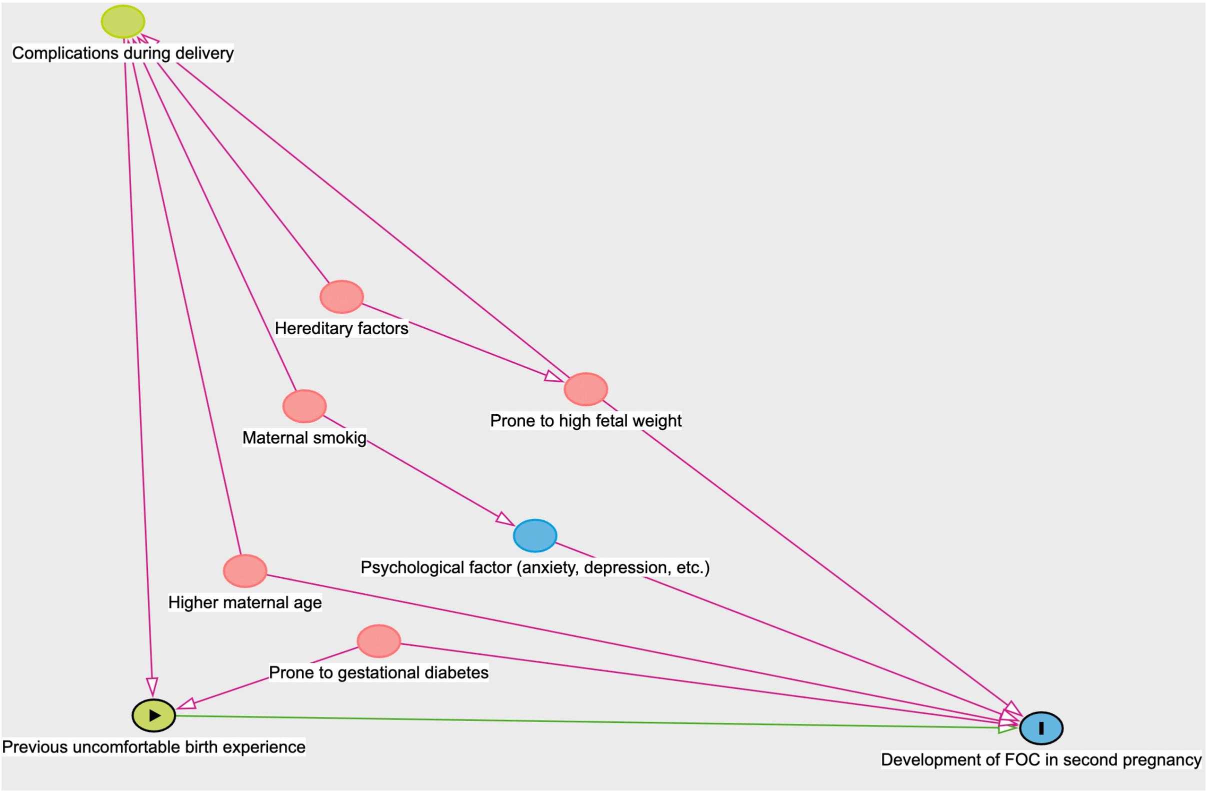

Supplementary Figure 1: Directed acyclic graph (DAG) on the logistic regression models used in this study. Delivery mode, obstetric challenge or adverse neonatal outcome in the first pregnancy, considered as an uncomfortable birth experience, were used as an exposure variable, and the risk for the development of fear of childbirth (FOC) was used in the second pregnancy as an outcome.
